# Adverse health effects of emerging contaminants on inflammatory bowel disease

**DOI:** 10.3389/fpubh.2023.1140786

**Published:** 2023-02-24

**Authors:** Xuejie Chen, Sidan Wang, Xueyi Mao, Xin Xiang, Shuyu Ye, Jie Chen, Angran Zhu, Yifei Meng, Xiya Yang, Shuyu Peng, Minzi Deng, Xiaoyan Wang

**Affiliations:** ^1^Department of Gastroenterology, The Third Xiangya Hospital of Central South University, Changsha, Hunan, China; ^2^Hunan Key Laboratory of Nonresolving Inflammation and Cancer, Changsha, Hunan, China; ^3^Xiangya School of Medicine, Central South University, Changsha, Hunan, China; ^4^Centre for Global Health, Zhejiang University, Hangzhou, China

**Keywords:** inflammatory bowel disease, emerging contaminant (EC), exposome, adverse health effects (AHEs), gut dysbiosis

## Abstract

Inflammatory bowel disease (IBD) is becoming increasingly prevalent with the improvement of people's living standards in recent years, especially in urban areas. The emerging environmental contaminant is a newly-proposed concept in the progress of industrialization and modernization, referring to synthetic chemicals that were not noticed or researched before, which may lead to many chronic diseases, including IBD. The emerging contaminants mainly include microplastics, endocrine-disrupting chemicals, chemical herbicides, heavy metals, and persisting organic pollutants. In this review, we summarize the adverse health effect of these emerging contaminants on humans and their relationships with IBD. Therefore, we can better understand the impact of these new emerging contaminants on IBD, minimize their exposures, and lower the future incidence of IBD.

## Introduction

Inflammatory bowel disease (IBD) refers to chronic, relapsing inflammatory disorders of the gastrointestinal tract, and its pathogenesis includes heredity and environmental factors (1). The two major types of IBD are Crohn's disease (CD) and ulcerative colitis (UC) (2). The pathology of IBD involves impairment of the intestinal mucosal barrier, dysbiosis of the gut microenvironment, and the alteration of the gut immune response (3). Chronic abdominal pain and diarrhea are typical symptoms of IBD. Presently no effective treatments can fully cure IBD, and nearly 30% of IBD patients will require surgery within 10 years after their initial diagnosis (4, 5). The global spread of IBD appears to be associated with industrialization and changes in people's diets and environments, and environmental exposures are closely associated with the increased risk of IBD (6).

**Figure 1:**
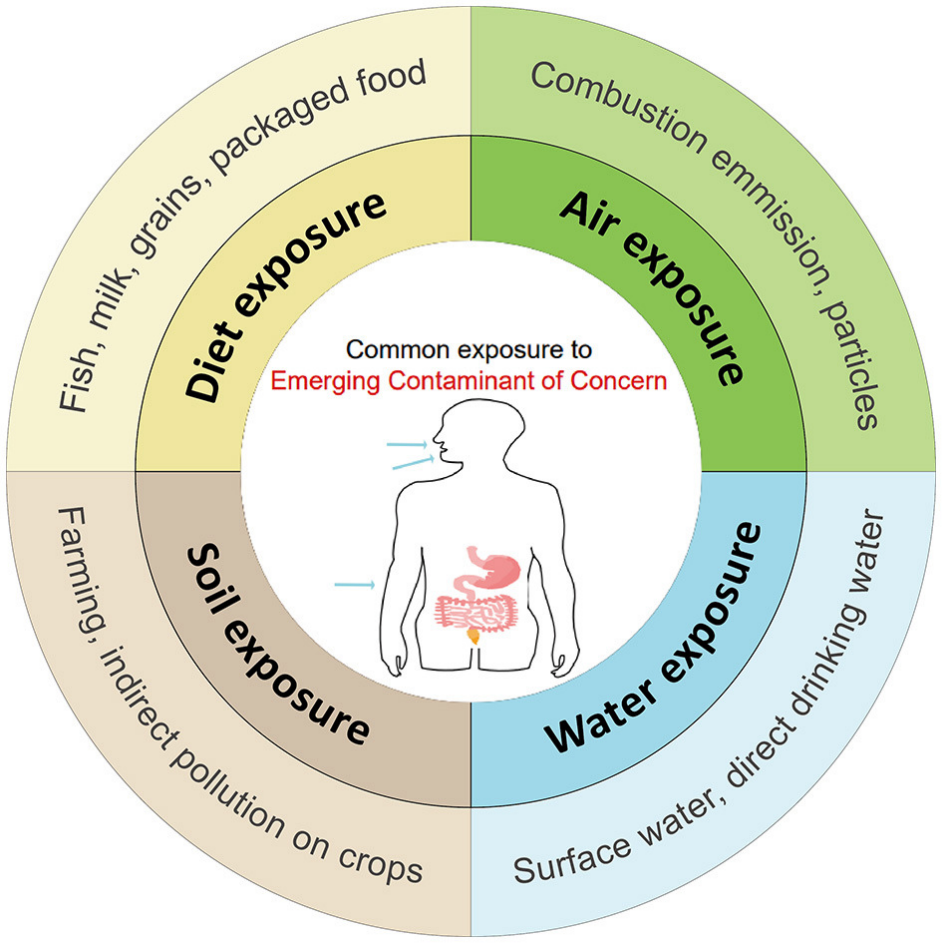
The common way and pollutants that general population in exposure of Emerging Contaminants (ECs) through digestive tract, respiratory tract and skin contact.

The new emerging environmental contaminant is a recently coined term that describes exposome to the environment. The emerging environmental contaminants including but are not limited to microplastics (MPs), endocrine-disrupting chemicals (EDCs), chemical herbicides, heavy metals, and persisting organic pollutants (POPs) (7, 8). The variable composition of these exposomes across regions, and the interaction among these exposures may contribute to the heterogeneous nature of the association between emerging environmental contaminants and IBD (9). These exposomes are not commonly monitored in nature, but have the potential to enter the environment and human body, and cause short-term and long-term adverse health effects. In the immediate dietary intake, the contaminants may cause acute abdominal pain or diarrhea, activating immediate intestinal inflammation (Figure 1). As in long-term exposure, these contaminants will cause chronic diseases like IBD and chronic renal failure, activate a series of chronic inflammation.

In this review, we summarize the current epidemiologic evidence and biological mechanism between new emerging contaminants exposure and the development of IBD. Also, we summarize the common exposure pathways of new emerging contaminants to public generations based on accumulated studies.

## Microplastics

Microplastics (MPs) are tiny plastic particles under 5 millimeters in size (10). The primary sources of MPs in human life are plastic bottles, abrasives, and opacifiers (11), and they may be degraded into MPs by various factors like ultraviolet over time (12). The main types of MPs include polyethylene (PE), polypropylene (PP), polystyrene (PS), polyethylene terephthalate (PET), polyvinyl chloride (PVC), Polyurethane (PU), etc. (10, 13) (Table 1).

**Table 1:** Adverse health effects of microplastics in inflammatory bowel disease.

**Contaminant**	**References**	**Experiment model/human group**	**Exposure time/dose**	**Route of exposure**	**Source of dietary intake**	**Impact on the gut**	**Other health risks**
Polystyrene (PS)	Schwarzfischer et al. (14)	Wild type female C57BL/6 mice	0.2 mg/day for 84 days	Dietary exposure	Drinking water	° Penetrate the intestinal barrier and accumulate in small intestine, lymphoid organs and liver ° Do not affect intestinal health, nor aggravate acute/chronic DSS colitis	/
	Luo et al. (15)	8-week-old male C57BL/6 J mice	0.5/5 μg for 14 days	Oral gavage	Drinking water	°↓Colonic length °↑Histopathological damage °↓Mucus secretion °↑Colon permeability °↑Colonic inflammatory response	°↑Secondary liver injury associated with inflammatory cell infiltration
	Zheng et al. (16)	male C57 mice	500 μg/L for 28 days	Dietary exposure	Drinking water	°↑Acute colitis and lipid disorders induced by sodium dextran sulfate (DSS) °↑intestinal permeability	° Intensify liver damage in mice with acute colitis ° Affect lipid metabolism
	Lu et al. (17)	Five-week-old mice	0.5 and 50 μm polystyrene MP, 100 and 1,000 μg/L, 5 weeks	Dietary exposure	Drinking water	°↓Decrease the secretion of mucin in gut ° Induce gut microbiota dysbiosis	° Induce hepatic lipid metabolism disorder in mice
Polyethylene (PE)	Li et al. (18)	5-week-old SPF grade male C57BL/6 mice	Respective 6, 60, or 600 μg daily for 5 weeks	Dietary exposure	Special feeds	° Induce intestinal dysbacteriosis °↑Gut microbial species, bacterial abundance and flora diversity ° Induce inflammation in intestine °↑the secretion of serum pro-inflammatory cytokine IL-1α; °↓the percentage of Th17 and Treg cells among CD4^+^ cells	/
Most Polypropylene (PP)	Schwabl et al. (19)	8 healthy volunteers aged 33 to 65 from Tokyo	7 days	Dietary exposure	Seafood; Food is usually wrapped, packaged or stored in plastic; Chewing gum	° Metastasize to gastrointestinal tissues or other organs and cause harmful effects ° Patients with increased intestinal permeability (e.g., IBD patients) may be more susceptible to microparticle absorption and potential damage.	/
	Zhang et al. (20)	26 healthy male students aged 18–25 from Beijing	8 days	Dietary intake	Packaged water, beverages, milk, dairy products, beer	/	° Moderate correlation exists between the intake of packaged water and the abundance of microplastics in feces.

“↑” means increased level or concentration; “↓” means decreased level or concentration.

Human exposures to MPs occur through ingestion, inhalation, and dermal contact (21). MPs exist and exposit in all environments, especially underground and surface water, then the human food chain, and ultimately enter the body through ingestion, which is the major exposure way (11, 13, 22, 23). A recent review has summarized multiple types of food containing MPs, including fruit, vegetables, milk, meat, aquatic food, etc. (12, 24). Besides, the fast spread of takeaway foods accelerates the number of MPs ingested by humans globally, since they are usually packed with plastic products (12). The suggested weekly ingestion range of MPs is within 0.10–5 g/week (5, 7, 8). In a study of a small set of donors, which first measured the mass concentration of the polymetric component of plastic in human blood, the mean of the sum quantifiable concentration of plastic particles in blood was 1.6 μg/ml (25). In human feces, MPs were also detected in the order of 2 MP particles/g (5).

MPs could be detected in the human bloodstream probably due to the absorption of them into the blood by mucosal contact (either ingestion or inhalation) in a size-dependent manner (25). MPs may be transported to organs *via* the bloodstream, causing intestinal toxicity, metabolic disruption, reproductive toxicity, neurotoxicity, immunotoxicity through oxidative stress, apoptosis, and specific pathways, etc. (26). After dietary intake, MPs are usually absorbed in the digestive tract, liver, kidneys, and spleen (27–31). Once deposited, MPs will induce morphological changes, activate inflammatory responses, inhibit cell differentiation, and affect gene expression (27, 32–34). Several clinical trials found that exposure to MPs impairs the gut epithelial barrier, induces intestinal flora alteration, disturbs lipid metabolism, causes oxidative stress and the release of inflammatory factors (15, 35–37). Apart from passive intake, the intestinal tract absorbs MPs through multiple ways, including endocytosis of enterocytes and specialized M cells, paracellular uptake, and active absorption by the intestinal villus (38).

The increased exposure to MPs will also accelerate the pathogenesis of IBD. A recent study revealed that exposure to MPs may impair the antimicrobial capacity of blood clam by reducing plasma inhibition of bacterial growth, humoral immune effector levels, and chemotactic activity of hemocytes, which showed that MPs imposed significant oxidative stress on hemocytes, causing great immunotoxicity (39).

Researchers found that the average concentrations of MPs in the feces of IBD patients (41.8 items/g dm) were markedly higher than that of healthy people (28.0 items/g dm) (40). In IBD patients, the gut experiences repeating endothelial lesions, which increases the permeability of the intestinal epithelial barrier (41). This means in IBD patients, MPs are more likely to enter the injured intestinal epithelial cells, attach to them, and enhance their translocation to different systems (14, 42). MPs exposure also triggers the over-proliferation of intestinal stem cells, causing the imbalance of colonic epithelial homeostasis, which in turn elevates the occurrence and severity of DSS-induced colitis (43).

MPs will also alter the composition and diversity of intestinal microflora in animal models, which will trigger multiple follow-up effects, such as changing the ability in the differentiation of Treg cells, and activating the signal transduction pathways associated with intestinal mucosal immune function (17, 18, 44–46). In the DSS mouse model, additional exposure to polystyrene microplastics (PS-MPs) can aggravate the severity of colitis (14, 15) by reducing tight junction proteins such as Claudin1 and Occludin, and increasing intestinal permeability (47–49). Correspondingly, TNF-α, IL-1β, and IL-6 increase significantly in the colitis mice exposed to additional PS-MPs, demonstrating their proinflammatory properties (15, 16). After the exposure, the macrophage infiltration will increase in the liver, which further triggers immune cells to release proinflammatory and immune factors like IL-17α and IL-22 (16).

MPs can alter the structure of intestinal microbiota in mice, which may exacerbate intestinal inflammation. Previous studies showed the overgrowth of Staphylococci is related to IBD. Correspondingly, mice treated with MPs exhibited a marked increase in Staphylococcus genus abundance and a decrease in Parabacteroides genus abundance (*P* < 0.05) (18). Li et al. observed increased numbers of gut microbial species, bacterial abundance, and flora diversity in mice treated with a high concentration of MPs (50). An increased abundance of Staphylococcus and Bacteroidetes alongside a decreased abundance of Parabacteroides and Firmicutes are also documented in this research (18). Likewise, Lu et al. found that MPs exposure decreased the abundances of Firmicutes and α-Proteobacteria in the feces of mice, and altered the variety and diversity of gut microbes (17).

To sum up, exposure to MPs, either in the blood or digestive tract, may accelerate the occurrence and development of IBD, and cause serious harm to the human body. Therefore, it is necessary to reduce the amount of dietary intake of MPs, such as reducing the use of bottled water and takeaway food (12, 23). The government also needs to pay attention to more efficient ways to degrade MPs. The potential benefits of reducing the pollution of MPs at sources still deserve further exploration.

## Endocrine disruptors

Endocrine disruptors (EDCs) are chemicals that interfere with the hormones in the human body through the endocrine system (51). EDCs can affect the way the body reacts to hormones, and change the gut microenvironment, which may cause immune vulnerability, decreased tolerance to food antigens (52), change in gut microbiota, and the occurrence of IBD (53).

EDCs, including phthalates, flame retardants, pharmaceutical agents, and phenols like bisphenol-A (BPA), ethyl-paraben, and methylparaben (54), are massively produced and used for food containers, personal care products, and other plastic objects (51). EDCs enter the human body mainly through dietary ingestion, inhalation, and dermal uptake, and are mostly bioaccumulated in the adipose tissue (55, 56). Most EDCs are lipophilic; therefore, they can induce microbial dysbiosis, and activate xenobiotic pathways and associated genes, enzymes, and metabolites (Table 2).

**Table 2:** Adverse health effect of EDCs in inflammatory bowel disease.

**Contaminant**	**References**	**Experiment model/Human group**	**Exposure time/dose**	**Route of exposure**	**Source of dietary intake**	**Impact on the gut**	**Other health risks**
PCBs	Min et al. (57)	Female C57BL/6 mice	5 mg/kg for 6 weeks	Oral gavage	PCB153 dissolved in corn oil	° Deteriorate the health of gut microbiota.	° Induce obesity, lipid metabolism disorder, and dyslipidemia.
BPA	Javurek et al. (58)	California mice (Peromyscus californium)	BPA (50 mg/kg) or EE (0.1 ppb)	Diet from periconception through weaning	Artificial feeding	° Increase gut microbiota proportions °↑Bacteroides, Prevotellaceae, Sutterella and etc.	° Disrupt normal gut flora; ° Induce IBD and colorectal cancer.
	Lai et al. (59)	Sixteen 3-week-old male CD-1 mice	Bisphenol A (>98% purity) solution (120 mg/mL)	BPA-water feed	Drinking water	° Reduce gut microbiota diversity ° Induction of Helicobacteraceae ° Reduction of Firmicutes and Clostridia.	° Alter the gut microbiota; ° Induce IBD.
	Yin et al. (60)	Seven-week-old DSS-induced colitis mouse: ICR mice	BPA and its substitute BHPF, 2 weeks	Dietary exposure	Not mentioned	° Induce inflammatory responses ° Alter gut metabolites ° Deregulate sugar and fatty acid metabolisms.	° Change the metabolic way of gut microbiota in IBD.
	Diamante et al. (61)	Eight-week-old female and male C57BL/6J mice, eight breeding pairs	0.005 μg/μL BPA and 0.0015% ethanol;	Exposed during gestation, terminated after 19–21 days	Artificial feeding	° Affect fatty acid metabolism, and the gut microbial composition.	° Affect the gut microbial composition in an age- and sex-dependent manner.
	Linares et al. (62)	200 CD patients (140 in remission; 60 in active disease)	/	/	Food in EDCs containers; packaged food, seafood.	° BPA: higher in colonic vs. ileal forms ° Butyrate and tryptophan: lower in exposed patients.	° Increased in serum of patients with active disease vs. patients in IBD remission period.

“↑” means increased level or concentration; “↓” means decreased level or concentration.

BPA is the most common and well-known EDC. It is linked with the active type of IBD. The direct intake of BPA-packaged food rarely causes an immediate intestinal response, as it mainly affects humans through chronic exposure and accumulation. BPA level is significantly increased in the serum of patients with the active period of IBD, compared to patients in the remission period, and regarding the disease phenotype, serum BPA levels were higher in colonic vs. ileal forms (62). It can change the level of microbiota and gut metabolites, increase the incidence of IBD, and accelerate IBD development (57, 59, 62).

BPA may change the metabolic way of gut microbiota and increase the risk of IBD. Many bacteria, such as Bacteroides, Mollicutes, Prevotellaceae, Erysipelotrichaceae, Akkermansia, Methanobrevibacter, and Sutterella, whose proportions increase with exposure to BPA, are associated with different diseases, such as IBD and colorectal cancer (58). Another American research shows BPA can affect the gut microbial composition in an age- and sex-dependent manner (61). In the epidemiological studies, dietary exposure to BPA reduces gut microbial diversity, and in the gut microbiota of CD patients, the population of Proteobacteria increased while Clostridium decreased (59). In the plasma of UC patients, the amount of BPA exposure is observed to have correlation with the altered level of plasma proteins involved in lipid-related metabolic processes and cytokine response, indicating BPA may serve as a biomarker in severe UC (63). BPA exposure may also change the level of gut metabolites, reducing butyric acid and tryptophan, and increasing fecal calprotectin, which indicates a correlation between its exposure and the severity of IBD (57, 62).

Aside from BPA and its analogs, many other EDCs also relate to the development of IBD, such as phthalates, triclosan, and perfluoroalkyl substances (64–66). For example, exposure to the BPA substitute, fluorene-9-bisphenol, can alter gut metabolites in mice and deregulates the sugar and fatty acid metabolisms in the gut (60). Several epidemiological studies have demonstrated the correlation between other EDCs and IBD (62, 66, 67). In a study estimating paraben and phenol exposure, approximately one quarter (25.5%) of all participants in this sub-set reported symptoms of chronic diarrhea, which is a typical complication of IBD (67). Notably, in IBD-specific cases, higher mean concentrations of urinary 4-tert-octylphenol were associated with the increased prevalence of IBD (67).

To reduce the adverse health effects of BPA, in 2015, the European Food Safety Authority (EFSA) reduced the tolerable daily intake (TDI) of BPA from 50 μg/kg body weight (bw)/d to 4 μg/kg bw/d (68). The estimated recommended daily intake of BPA ranges from < 1 to 5 μg/kg bw/d (69). To reduce EDCs exposure, it is essential to choose EDCs-free products and not heat food or store hot food in BPA containers marked with the recycling code 3 or 7 (70). From the present situation, it is important to strengthen the food safety policy, and use suitable materials in direct contact with food (56, 71). In the meantime, the necessary action is to reduce waste and use EDCs-free packaging, which may contribute to health improvement in food and reduce the risk of IBD.

## Chemical herbicides

Chemical herbicides are herbicides that inhibit the growth of unwanted plants like residential weeds and invasive species (72). Commercially used chemical herbicides can cause substantial mortality of non-target plants and insects. They contaminate soil and reside in water, and may accumulate in the environment over time (Table 3). Glyphosate is the most popular herbicide in America. Its concentration continued to soar in the world, with the level rising from 2 to 430 μg/L in natural water (79). The rising level in water causes severe pollution and threatens food safety.

**Table 3:** Adverse health effect of chemical herbicides in inflammatory bowel disease.

**Contaminant**	**References**	**Experiment model/human group**	**Exposure time/dose**	**Route of exposure**	**Source of dietary intake**	**Impact on the gut**	**Other health risks**
Glyphosate	Tang et al. (73)	Eight-week-old male Sprague rats, weighing 180–220 g	0, 5, 50, and 500 mg/kg of weight glyphosate, for 35 days	Oral gavage	Artificial feeding	°↓The relative of Lactobacillus in small intestine	° Induce inflammatory responses ° Alter gut microbial composition
	Suppa et al. (74)	Model species Daphnia	1 mg/L glyphosate, corresponding to the MCL of drinking water	Water surroundings	Not mentioned	° Induce DNA damage ° Alter the gut microbiota ° Interfere carbon and fat metabolism	° Dysbiosis of gut and its chronic inflammation
	Ding et al. (75)	Six-month-old healthy adult male zebrafish (Danio rerio, AB-wild type)	3.5 mg/L GLY concentration	Water surroundings	Not mentioned	° Alter the gut microbiota °↑Fusobacteria ↓Proteobacteria °↓Claudin-5, ZO-1, occludin	° Gut dysbiosis ° Destroy the intestinal mucosal barrier ° Enhanced intestinal permeability.
2,4-D	Tu et al. (76)	Specific-pathogen-free (SPF) 8-week-old C57BL/6 male mice	Low-dose 2,4-D exposure: 1 ppm 2,4-D water solution, 13 weeks	Feeding water solution daily	Artificial feeding	° Influence the homeostasis of gut microbiome °↓ Plasma acylcarnitine.	° Alter microbiome-related pathways ° Disturb gut-host homeostasis ° Increase risk of IBD
Combination of 2,4-D, dicamba and glyphosate	Mesnage et al. (77)	Wild-type mES cells (strain B4418)	The mixture of glyphosate, Dicamba and 2,4-D in water.	Water surroundings	/	° DNA damage ° Oxidative stress ° Unfolded protein response	° Carcinogenic effects
Propyzamide	Sanmarcro et al. (78)	Zebrafish (7 d.p.f.)	Immersed in TNBS-containing E3 ° Medium for 72 h.	Liquid surroundings	/	° Upregulate NF-κB-driven C/EBPβ proinflammatory gene expression ° Inhibit AHR signaling ° Boost intestinal inflammation	° Increase the risk of IBD and other gut diseases

“↑” means increased level or concentration; “↓” means decreased level or concentration.

Chemical herbicides affect humans mainly through intestinal absorption, skin exposure, and inhalation (69). They may enter the body through contaminated food, like crops, fruits, and vegetables (80). In a German survey, the urinary glyphosate concentrations of 3 to 17-year-old children were above the limit of quantification of 0.1 μg/L, and the overall exposure of the young population may relate to their vegetarian diet or consumption of cereals, pulses, or vegetables (81). Much research hasproven the adverse effects of chemical herbicides on non-human beings, such as mice, zebrafish, and livestock.

Herbicides, including glyphosate, 2,4-Dicholorophenoxy acetic acid (2,4-D), and dicamba, may damage the immune system and cause symptoms of IBD, like diarrhea, bowel inflammation, and maldigestion (77). Especially, people with weakened immune systems are more susceptible to chemical herbicide-related intestinal inflammation (82). Herbicides may disrupt the normal gut flora, and irritate the lining of the digestive tract, which can lead to gut inflammation and gastrointestinal diseases like IBD (83).

Glyphosate is an active ingredient in Roundup, the most widely-used herbicide (84, 85). Glyphosate exposure may be a critical environmental trigger in the etiology of diseases associated with gut microbiota dysbiosis, including IBD (73, 86, 87). Glyphosate can alter the structure of microbiota, interfere with the shikimate pathway in microbiomes, and hinder the production of aromatic amino acids (88, 89). And the dietary intake of aromatic amino acids may alleviate the antimicrobial effect of glyphosate (90). Glyphosate exposure induces inflammatory responses in the small intestine, and alters the gut microbial composition in rats, with the *Lactobacillus* significantly decreasing (73). It also destroys the intestinal mucosal barrier function, leading to dysbiosis and chronic inflammation.

Other essential elements in chemical herbicides include dicamba, 2,4-D, 2,4-Dinitrofluorobenzene, and 2,4,5-Trichlorophenoxyacetic acid. The sub-chronic low-dose 2,4-D exposure may influence gut microbiome homeostasis, significantly lower the acylcarnitine level, and decrease levels of plasma acylcarnitine (76). An IBD multi-omics research showed that the rising level of acylcarnitine is closely related to the development of IBD (91). But another explanation is that decreasing acylcarnitine levels can also produce toxicity, suggesting the perturbations in the fatty acid beta-oxidation pathway (92). Dicamba, 2,4-D, and glyphosate alone or in combination, account for genotoxicity in patients with gastrointestinal disorders, including DNA damage, oxidative stress, and unfolded protein response, which contributes to the development of IBD (77). Another herbicide, Propyzamide, can boost gut inflammation by upregulating NF-κB-driven C/EBPβ pro-inflammatory gene and inhibiting AHR signaling pathways, and further, inducing the development of IBD (78). Furthermore, an organic diet can reduce the human body's herbicide level, which is a possible solution to reduce herbicide residue and lower the risk of IBD.

## Heavy metals

Heavy metals are naturally occurring elements with high atomic weight and density (93). They are also called trace elements, usually detected in trace concentrations (ppb range to < 10 ppm) (94). The heavy metals we discuss are those accumulated in the food chain and are highly toxic to living organisms. Most of them come from natural resources and industry (95–98), including lead (Pb), manganese (Mn), arsenic (As), cadmium (Cd), mercury (Hg), and others. They are commonly used in people's daily life with widespread pollution (99, 100).

Human activities may increase the number of heavy metals residing in the environment, including metal processing, and the production of medical waste, plastic products, and electric wastes (101–104). Dietary is the main source of human exposure, with its detrimental effects including cardiovascular, neurological, reproductive, and intestinal disorders (105–108). Here we focus on five heavy metals: Pb, Mn, As, Cd, and Hg. They are not only ubiquitous in the environment, but also associated with gut microbiota dysbiosis and the severity of IBD (Table 4).

**Table 4:** Adverse health effect of heavy metals in inflammatory bowel disease.

**Contaminant**	**References**	**Experiment model/human group**	**Exposure time/dose**	**Route of exposure**	**Source of dietary intake**	**Impact on the gut**	**Other health risks**
Lead	Yu et al. (11)	C57BL/6 mouse models	0.1, 0.5, and 1.0 g/L for 8 weeks	Dietary exposure	Drinking water	°↓ Expression of tight junction proteins °↑ Abundance of Marvinbryantia and Ruminococcus °↓Abundance of Lactobacillus and Roseburia ° Induce gut dysbiosis	° Influence the metabolism of macronutrients, trace elements ° Neurodegenerative injury ° Inhibit CAT activity in kidney and GSH level in liver
	Xia et al. (109)	Male adult wide type AB strain zebrafish (*Danio rerio*)	Respective 10 and 30 μg/L for 7 days	/	/	°↑ Gut mucus volume °↓The abundance of α-Proteobacteria °↑ The abundance of Firmicutes	° Induce hepatic metabolic disorder
Manganese	Choi et al. (110)	Wild type C57BL/6 mice aged 3–4 weeks	Mn: 0–0.5 ppm (deficient), 35–35.5 ppm (adequate), and 300-301 ppm (supplemented).	Dietary exposure	Diets	° Maintain the intestinal barrier °↑ Morbidity, weight loss, and colon damage °↑ Levels of inflammatory cytokines	/
	Mitchell et al. (111)	Male C57BL/6 mice	MnCl_2_ (66 mg/kg)	i.p. injection	/	° Reduce chronic colitis	/
Arsenic	Zhong et al. (112)	1-day-old ducks	control group; low ATO group 4 mg/kg; high ATO group 8 mg/kg.	Oral administration and intubation	Drinking water	° Intestinal injury °↓α diversity of intestinal flora ° Change bacterial composition °↓ Expression of intestinal barrier related proteins	° Liver inflammatory cell infiltration ° Vesicle steatosis °↑ Pro-inflammatory CKs (IFN-γ TNF-α IL-18 and IL-1β) in the liver
Cadmium	Breton et al. (113)	12-week-old female BALB/c mice	CdCl_2_ (2.5 and 12.5 mg/kg) for 1, 4, or 6 weeks	Dietary exposure	Drinking water	°↓ Epithelial permeability °↑ Oxidative defense mechanism °↓ Nf-κB and pro-inflammatory cytokine pathways ° Stimulate anti-oxidant pathways	/
Mercury	Zhao et al. (114)	Female Kunming mice	80 mg/L HgCl_2_ for 90 days	Dietary exposure	Drinking water	↑ Faecalis, Helicobacter °↓ Halococcus and Bacillus ° intestinal injury	↑ Pro-apoptotic gene expression
	Zhao et al. (115)	Eight-week-old female mice	HgCl_2_ (160 mg/L) for 3 days	Dietary intake	Drinking water	↓ Growth performance ° Induce oxidative stress °↑ Clostridium, Lactobacillus	/
	Seki et al. (116)	Female C57BL/6 mice	MeHg (5 mg/kg) for 14 days	Oral intubation	/	° Inhibit the growth of lactobacillus	°↓ Gut bacteria after exposure to methylmercury ° Accelerated accumulation in the cerebellum, liver, and lungs

“↑” means increased level or concentration; “↓” means decreased level or concentration.

### Lead

Most Pb emissions in life come from gasoline and enter the environment through burning exhausts. Pb enters the body mainly through dietary exposure, including food (65%) and water (20%) (117). For the Pb in the food, adults can absorb 10–15% of the ingested Pb, while children can absorb up to 50% through the gastrointestinal tract, which indicates children are more susceptible to Pb exposure.

Most Pb ingested accumulates in the kidneys, followed by the liver and other soft organs, like the heart and brain (94). When its dose is over 70 μg/dL, severe consequence happens (118). Pb can cause various disorders by inducing oxidative stress and breaking membrane integrity (119, 120). It also impairs gastrointestinal function and contributes to IBD pathogenies.

Epidemiological findings show that the level of Pb in IBD patients rises significantly (121). In CD patients, Pb in the scalp hair were significantly lower than that in healthy individuals, and its concentration in the serum is lower, which is consistent with the rising level in the tissue (122). In animals exposed to Pb, the amount of intestinal mucus increases, and the diversity and abundance of gut microbiota also change significantly (123, 124). Many metabolites related to glucolipid metabolism, amino acid metabolism, and nucleotide metabolism have changed after Pb exposure (109). Besides, Pb is highly toxic to Escherichia coli and Lactic acid bacteria, and long-term Pb exposure will induce chronic toxicity in a dose-dependent manner (11).

Developed countries have higher centration of Pb emission (125), which corresponds to the fact that IBD in developed countries shows a higher prevalence. Therefore, it is necessary for people in developed countries to take more precautions.

### Manganese

Manganese (Mn) is the 12th most abundant element on the Earth. It exists mainly in the chemical oxidation state (126), and it is necessary for normal body functioning (94). It can activate various enzymes in the body and is indispensable for the development of intestinal immune functions (126).

The content of Mn in vegetables is higher than that of animal food (127). Seafood, chocolate, nuts, fruits, rice, and spices are also essential sources of Mn (127). The average concentration of Mn in human tissue is 1 mg/kg (126). Excessive dietary intake may lead to impaired intestinal immune function and over-activate oxidative stress, which is closely associated with the inflammation process in IBD patients.

The serum Mn concentration is different between healthy individuals and IBD patients, with the extent much greater in IBD patients (121, 128, 129). The concentration of Mn in blood is markedly higher in CD patients (121). But animal experiments showed some contradictions. In Mn-deficient mice treated with DSS, the incidence of IBD increases, along with higher inflammatory cytokine levels, oxidative damage, and DNA damage, which indicates the level of Mn may be inversely correlated to the incidence of IBD.

Another two studies also indicate Mn's protective role in gut homeostasis. One shows that a decrease in absorption and accumulation of Mn will trigger the release of proinflammatory factors and exacerbate the severity of inflammation (111). Another study implies Mn can boost the immune system by enhancing the function of intestinal CD8+ T cells (130). Furthermore, some studies have indicated the protective role of Mn for IBD. A study on manganese metal-organic framework (Mn-MOF), is a practical application in the treatment of spontaneous IBD by scavenging ROS to relieve oxidative stress, and protect the intestinal barrier (131). The study on hollow MnO_2_ (hMnO_2_) carried out to achieve synergistic IBD therapy, is based on MnO_2_, which has highlighted SOD-like and CAT-like activities (132, 133). According to the aforementioned viewpoints, the role of Mn for IBD is contradictory. For IBD patients, higher serum Mn concentration may be a protective reaction to avoid producing rapid and excessive ROS. The assumption can be consistent with the fact that IBD is a chronic disease. Experimenting to test the changes in serum Mn concentration with time is valuable. These exposure may increase the risk of IBD through oxidative stress and other mechanism in the gut. It is necessary to come up with the hypotheses to sort out the contradiction.

### Arsenic

Arsenic (As) is an essential and poisonous substance commonly found in contaminated soils and water (134). It is also rich in fish and marine mollusks (135). The roots of crops and vegetables also contain high-concentration As (118).

According to World Health Organization, the permissible limit for As in drinking water is 10 μg/L (136). And if the exposure dose is over 50 μg/L, it can lead to gastrointestinal tract dysfunction and multiple organ disorder (136–139). Long-term exposure or high ingestion doses may increase the accumulated As in the gut (140).

Mounting evidence has revealed some intrinsic connections between As exposure and IBD. It enters the body through dietary intake, and metabolites to arsenic trioxide (ATO) in the gut, which is toxic to the gut microenvironment (112). It not only induces intestinal damage and liver inflammatory cell infiltration, but also reduces gut microbiota diversity (112, 141). In ATO exposure, the expression of intestinal barrier-related proteins, such as Claudin-1, MUC2, ZO-1, and occludin, significantly decreases, resulting in increased intestinal permeability (142, 143). However, with exposure to inorganic As, the expression of Claudin-1 reduces, resulting in increased permeability and intestinal barrier disruption. ATO can also activate inflammasome NLRP3, and induce a cascade effect of the LPS/TLR4/NF-κB signaling pathway, which exacerbates the inflammatory severity (112). However, ATO can inhibit NF-κB expression, increase procaspase-3, and induce caspase-3 activation leading to apoptosis to eliminate inflamed cells, which indicates the anti-inflammatory effect of ATO. Another study shows that ATO exposure can alleviate the inflammatory extent in DSS-induced IBD mice by increasing catalase and GSH levels to enhance antioxidants (144). In the epidemiological study, the level of serum As concentration is higher in CD patients compared to healthy adults (121). The causes for the distinct results may include the various metabolism of As in different species, exposure to the diverse form of As (inorganic As or ATO), the various regulation approaches, and the dose of As.

### Cadmium

Cadmium (Cd) is mainly used as an anticorrosion agent, and it naturally occurs in ores (93). Cd can enter the human body through contaminated food and water *via* the gastrointestinal tract, inhalation, and dermal tissues (145). Food is the most important source of Cd exposure in the general non-smoking population, which indicates the risk of dietary exposure. Recent studies have found that Cd is highly enriched in some aquatic animals like zebrafish and crabs. Ingestion of Cd is highly related to gastrointestinal disturbances such as diarrhea, nausea, and abdominal pain. Meanwhile, its chronic exposure can increase risks concerning multiple organ dysfunction, bone deformation, and contribute to cancer cell progression (146).

Cd exposure can also significantly affect the gut microenvironment. It can perturb the diversity and abundance of gut microflora, especially decreasing the number of Firmicutes and γ-proteobacteria (145). In addition, Cd exposure also elevates the level of TNF-α, IFN-γ, and IL-17 in the colon (147). Additionally, Cd exposure can increase intestinal permeability through decreasing mRNA expressions of ZO-1, ZO-2, occludin, and claudin-1 in the jejunum and colon, accompanied by intestinal histological changes (148).

However, research also indicates the potential protective role of Cd. Cd may interfere with LPS signaling, particularly disrupting macrophage inflammation by inhibiting the NF-κB pathway in the gut, inhibiting the pro-inflammatory effect of M1 macrophage (149). Short-term exposure to Cd exacerbates the symptoms of acute DSS- and TNBS-induced colitis, while sub-chronic exposure to Cd significantly alleviates some symptoms in DSS-induced colitis and reduces the severity of colitis in a dose-dependent manner. Its potential mechanisms include reversible reduction in epithelial permeability, stimulation of anti-oxidant pathways, upregulation of oxidative defense mechanism, and downregulation of Nf-κB and pro-inflammatory cytokine pathways (113). Moreover, the study also implies that the outcomes of Cd exposure may vary as a function of dose and exposure time. Along with other common heavy metals like Mn, As, and Pb, the Cd concentration is markedly higher in CD patients (121). Thus, further studies concerning Cd exposure relationship with IBD are needed to clear out the Cd dose, exposure time, and the synthetic effect of Cd regulating in different pathways.

### Mercury

Mercury (Hg) is a well-known component in medical apparatus like thermometers and other medical instruments (93). The absorptivity of Hg is 8–15% in the gastrointestinal tract (104). Human's primary exposure to Hg is dietary intake (125, 150–152).

Hg can accumulate in untreated wastewater from factory and agricultural runoff, which directly contaminates the crops and fish. Correspondingly, methyl mercury, a chemical substance converted from Hg, is discovered to be highly enriched in vegetables and fish, which implies the ability of Hg to accumulate in the food chain.

The toxicity of Hg can induce multiple organ failures when the dose of Hg exceeds 10 μg/L in blood or 20 μg/L in urine, such as lung injury, intestinal damage, proteinuria, allergies, and chronic poisoning.

Exposure to Hg is closely associated with IBD. Methyl mercury can accumulate in organs, and change the composition of gut microbes (116). Moreover, dietary exposure to Hg affects the growth of mice, partly due to changed gut microbiota (114). Mice exposed to Hg have a decreased abundance of Bacteroidetes and Proteobacteria, and an increased abundance of Clostridium, Lactobacillus, Treponema, and Helicobacter in the gut (115). The toxicity of Hg may also contribute to the development of IBD (115). It can directly break the calcium homeostasis and activate multiple enzymes by affecting the electron transport chains in mitochondria, producing superfluous reactive oxygen species (ROS) (115, 153, 154). Besides, ROS promotes mitosis, polyploid aberration, and susceptibility to DNA damage in the gut (155–157). Epidemiological studies have also shown altered enzyme activity in people exposed to Hg (29).

In conclusion, diet exposure is the common exposure route for heavy metals. Along with the food chain, heavy metals enter the human body, generating adverse health effects by various mechanisms. Gut injury caused by heavy metals is highly associated with the occurrence and development of IBD. Heavy metals can alter the gut microbiota by increasing some flora and decreasing other flora, then causing gut dysbiosis. In addition, some uncommon impacts include damaged intestinal barrier function, increased levels of inflammation cytokines, oxidative stress, etc. Meanwhile, other organ dysfunction can occur due to heavy metals exposure and accumulation. As heavy metals tend to accumulate in fish (107), people with a disease or hypo-immunity should reduce their eating frequency. And IBD patients should avoid dietary exposure to heavy metals. For relevant authorities, they should supervise factories' proper treatment of sewage and sludge to reduce heavy metals accumulation in crops (158–160). Bioremediation can also work by changing pollutants into food and energy (161, 162). Some novel ways also focus on dealing with heavy metal contamination, such as Particle Capture Systems, soil displacement/isolation, and Soil-flow-electrode capacitive deionization.

## Persistent organic pollutants

Persistent organic pollutants (POPs) are chemicals of global concern with the potential to persist in the environment. They can bio-accumulate and bio-magnify in ecosystems and threaten human health (71). POPs mainly include new pesticides, chemicals, and by-products of industrial production, which may lead to multiple effects on immune response and alter gut function (Table 5).

**Table 5:** Adverse health effects of POPs in inflammatory bowel disease.

**Study**	**References**	**Experiment model/human group**	**Exposure time/dose**	**Route of exposure**	**Source of dietary intake**	**Impact on the gut**	**Other health risks**
**New pesticide**							
Chlorpyrifos (CPF**)**	Huang et al. (163)	Eight-week-old DSS-induced male C57BL/6 mice	AIN-93 diet at doses of 1, 2.5, or 5 mg/kg/day CPF,6 days	Diet exposure	Artificial feeding	° Affect immune-cell populations °↑Inflammatory responses	° Lead to severe tissue injury ° Exert adverse effect on the gut microenvironment
Imidacloprid (IMI)	Luo et al. (164)	Adult male zebrafish	IMI (100 and 1,000 μg/L) in water solution, 3 weeks	Water surroundings	Not mentioned	°↑Superoxide dismutase and catalase (CAT) levels °↑LPS levels and inflammatory factors	° Cause intestinal barrier injury, oxidative stress, inflammatory response and gut microbiota dysbiosis
	Fu et al. (165)	The Pacific white shrimp *L. vannamei*	IMI (50, 100, 200, 400, and 800 μg/L) in water solution, 28 days	Water surroundings	Not mentioned	° Reshape the structure and interaction of gut microbiota °↑Gut pathogenic microbiota abundance ° Function disorders	°↓Growth performance ° Gause tissue damage in shrimp ° Cause disorder of differential gene expression
**Chemicals and by-products of industrial production**							
TCDD, TCDF, and PCBs	Tian et al. (166)	Cecal contents isolated from 7-week-old C57BL/6 J male wild type mice	TCDD (0.6, 0.06 μM), TCDF and PCBs (6, 0.6 μM), 37°C for 4 h	Direct contact: incubation	/	°↓Metabolic activity (dose-dependent) °↑Low nucleic acid (LNA) bacteria °↓High nucleic acid (HNA) bacteria	° Alter transcriptional and metabolic pathways in cecal bacterial mixtures ° Affect the physiological metabolism of individual bacteria
PBDD/**F**	Fernandes and Falandysz (167)	/	Some groups, particularly young children, may exceed the tolerable limit (2 pg TEQ/kg bw/week)	Present not only in soil, but also in plant foliage; Accumulate in the food chain	Mainly from dietary intakes: plant-based foods contain more PBDD/Fs	/	° Bind to the AhR, having carcinogenesis, immunotoxicity, enzyme induction and reproductive effects
TCDD	Li et al. (168)	C57BL/6 mice, 8–10 weeks old	0.1 and 10 μg/kg bw TCDD on embryonic day 0.5, ED 12.5, and post-natal 7 days	Oral gavage	Dissolved in dimethyl sulfoxide (DMSO) and diluted in olive oil	° Affect the structure and composition of the colonic microbiota ° Do not change the community diversity and richness ° Change the functional pathways of the colonic microbiota	/

“↑” means increased level or concentration; “↓” means decreased level or concentration.

### New pesticide

New pesticide is widely used to wipe out indoor and outdoor pests, such as imidacloprid, pyrethroids, and β-ketonitrile derivatives (169). It harms humans by taking the contaminated food and water (170), and causing intoxication through its accumulation in the food chain (171). Its exposure mainly includes the intake of vegetables, fruits, and grains. Among these, pesticide can residue more easily in grains. A food survey of Swedish adolescents showed that secondary school students who consumed grains had a higher exposure to pesticides than those who consumed vegetables and fruits, lending support to the findings (172).

Emerging links between pesticide residue and changes in the gut have emerged in recent years (173). Exposure to low-dose pesticide in diet seldom causes immediate health effect. Long-term exposure to chemicals in the pesticide can induce gut microbiota dysbiosis, alter the immune response in the gut, and contribute to the development of IBD (174, 175). Research on dietary exposure to chlorpyrifos, a widely used pesticide, suggests that dietary exposure can affect the population of immune-cell, induce inflammatory responses, and lead to severe tissue injury in DSS-induced colitis mice.

Another popular pesticide, imidacloprid (IMI), also adversely affects the gut microbiota (164, 165). IMI exposure can induce intestinal injury and oxidative stress in the gut of zebrafish (164). Additionally, it also results in a higher intestinal LPS level and the overexpression of inflammatory factors in the gut, as well as a rising level of the biochemical responses, transcriptome, and gut microbiota in the Pacific white shrimp. Human exposure to IMI is also observed in recent years. Currently, the maximum estimated daily intake of IMI [34.8 μg/kg bw/d] was lower than the chronic reference dose of IMI (57 μg/kg-bw/d) recommended by the United States Environmental Protection Agency (176). But IMI is already reported to have adverse effects on human semen quality parameters and the activation of macrophages in the body, which may increase the permeability of the intestine and impair the immune system (177, 178).

## Chemicals and by-products of industrial production

Another type of POPs is chemicals and by-products derivate from industrial production (179). These pollutants include Polychlorinated biphenyls (PCBs), Polybrominated dibenzo-p-dioxins and furans (PBDD/Fs), 2,3,7,8-Tetrachlorodibenzo-p-dioxin (TCDD), etc. (167). They can accumulate in the environment and exert a long-term adverse effect on human health (180). The food chain and the food web are the primary pathways of human exposure (180). Human exposure to them in diets mainly includes ingesting contaminated food, like fruits, vegetables, and grains, and eating polluted meat, milk, eggs, and fish, which is closely associated with IBD.

### Polychlorinated biphenyls

Polychlorinated biphenyls (PCBs) are synthetic organochlorine chemicals, which are mixtures of 209 different components (181). And PCBs are among the 12 initial POPs listed under the Stockholm Convention.

PCBs are mainly formed as by-products in manufacturing industries (182). They can reserve in soil and transfer to water surroundings, increasing the risk of human exposure *via* food chains (183, 184). Since PCBs are lipid-soluble, people who frequently eat animal fats can easily access PCBs (185). Contaminated meat and milk also show high concentrations of PCBs (186), and aquatic product consumption also increases the risk of PCBs exposure (181).

PCBs play a pro-inflammatory role in various diseases, which adversely affect IBD. They can induce oxidative stress by uncoupling CYP1A1 dose-dependently, and disrupt the normal endothelial barrier function (110, 187). Meanwhile, PCBs induce proinflammatory factors like IL-6 and vascular adhesion molecules such as VCAM-1, and then activate the NF-κB pathway (187–189). The expression of these molecules facilitates the recognition and migration of leukocytes, which are critical events of inflammatory responses (187). When exposed to PCBs, hosts can show disorders of gut microbiota, with reduced gut microbial diversity and variety (190, 191). In mice exposed to PCBs, the amount of Bacteroidales, Erysipelotrichales, Lactobacillales, Bifidobacteriales, Phyla Proteobacteria, Actinobacteria, Saccharibacteria, Deferribacteres, Firmicutes, and Verrucomicrobia increases significantly, while the level of Bacteroidetes decreases. Research in humans also shows that exposure to PCBs may interfere with the DNA hypomethylation of peripheral blood monocytes, inducing chronic inflammation (192).

### PBDD/Fs and TCDD

Apart from PCBs, other POPs such as PBDD/Fs and TCDD are also related to IBD (193). These organics are by-products of industry, which are classified into unintentional POPs (194). Their primary exposure pathway is dietary intake.

Plant-based food is reported to show higher PBDD/F, and the overly dietary intakes of PBDD/F suggest some population groups, particularly young children, will exceed the tolerable weekly intake (2 pg TEQ/kg bw/week) (167). The metabolic mechanism of PBDD includes causing oxidative stress, apoptosis, and cell damage. It can induce gut inflammation and dysbiosis of the gut microenvironment, leading to IBD development.

TCDD is the most potent chemical carcinogen evaluated by the US Environmental Protection Agency (195). It has a long half-life of 5–10 years in humans, due to its high lipophilicity and low metabolism (195). Most TCDD released into the atmosphere eventually settles onto the plant, soil, and water surfaces. After being taken, it accumulates in blood serum and adipose tissue, which leads to further damage in the body (195). An animal experiment showed that maternal exposure to TCDD suppresses the differentiation of Type 3 innate lymphoid cells (ILC3s) in the offspring, and distinctly affects colonic ILC3 function (196). Since ILC3s play a significant role in the mucosal immune response in the pathogenesis of IBD, there is a close connection between TCDD exposure and the occurrence and development of IBD (197). Moreover, TCDD can impact the gut microbiota and metabolic pathways, such as upregulating harmful bacteria and downregulating beneficial bacteria (168).

In sum, people are exposed to POPs primarily through dietary intake. POPs can alter transcriptional and metabolic pathways in cecal bacterial mixtures, modify gut microbiota-host homeostasis, and affect the metabolism of individual bacteria *in vitro* (166). POPs can also alter the microbial community structure and metabolic activities, leading to host disorders (198). Some POPs can increase the amount of Proteus and proportion of Firmicutes/Bacteroidetes, and increase the synthesis of short-chain fatty acid, which is related to the inflammatory changes of IBD (191). Epidemiological studies also present the adverse health effects of POPs. The interplays between POPs and gut microbiota lead to intestinal inflammatory changes and resultant toxicity (198). That is to say, the variations in the microbial communities partially indicate the body's exposure to these pollutants. Therefore, it is essential to formulate some daily dietary interventions such as prebiotics, probiotics, or symbiotics, which could impede or alleviate detrimental impacts induced by exposure to POPs.

## Conclusion

Mounting evidence of the underlying hazards of emerging contaminant exposures in IBD arouses increasing attention. However, the correlation of timing and frequency of such contaminant exposures to IBD incidence and disease phenotype was not specified. Environmental exposure may contribute to the pathogenesis of IBD through diet intake and the metabolic mechanism in the body. The currently accepted pathogenesis of IBD includes the interplay between genetic susceptibility and environmental factors, as well as the gut microbiome and the immune system.

The further relationship between these emerging contaminants and the risk of IBD deserves everyone's attention. Currently, these contaminants mainly exert long-term adverse effects by accumulating in the body and inducing chronic inflammation. Research in this field should focus more on the direct relationship and mechanism of these contaminants and the health of the human gut. And more epidemiological research about new emerging contaminants and their adverse health effects is needed.

**Figure 2:**
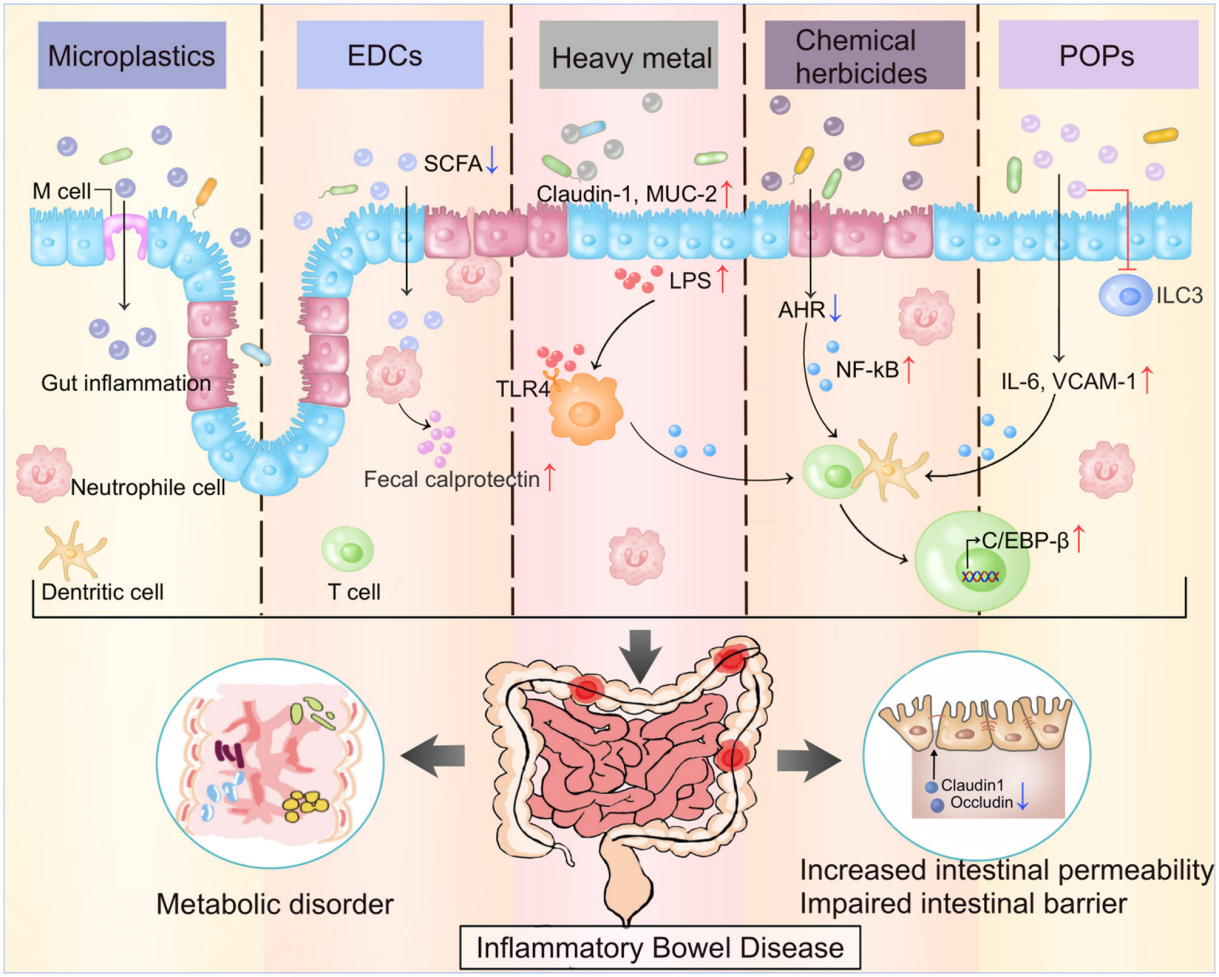
The main ECs and their impacts on gut microenvironment. ECs damage the intestine by regulating the intensity of immune response, releasing proinflammatory factors, impairing the intestinal barrier, and increasing the intestinal permeability through various mechanism. The figure illustrates the potential mechanism of five ECs mentioned in the review. ECs, Emerging contaminants; EDCs, Endocrine-disrupting chemicals; POPs, Persistent organic pollutants; SCFA, Short-chain fatty acid; TLR4, Toll-like receptors 4; ILC-3, Innate lymphoid cells; VCAM 1, Vascular cell adhesion molecule 1.

In this review, we have summarized the standard ways of exposure and inclusion of emerging contaminants, and their adverse effects on IBD patients through various underlying mechanisms (Figure 2). Exposure to these pollutants will increase the risk of IBD in healthy individuals. And people with IBD should pay special attention to preventing daily exposure to these contaminants, as they may cause adverse health effects regardless of age and exposure time. Exposure to these new emerging contaminants may increase the risk of IBD and accelerate the process of IBD. Therefore, understanding the role of these contaminants, including how they enter the body, how they induce immune-related reactions, and how they affect certain inflammatory diseases like IBD, will enable the more comprehensive formation of policies concerning the prevention and control, and will reduce medical expenses and burdens on the families and countries.

## Author contributions

XC: conceptualization, design, draft writing, and writing—review and editing. SW: design, draft writing, review, and writing—review and editing. XM: draft writing, review, and visualization. XX, AZ, YM, XY, and SP: methodology and review. SY: review and visualization. JC: methodology and writing—review and editing. XW: conceptualization, funding acquisition, and writing—review and editing. MD: conceptualization, design, methodology, and writing—review and editing. All authors contributed to the article and approved the submitted version.
